# Disease duration in autosomal dominant familial Alzheimer disease: A survival analysis

**DOI:** 10.1212/NXG.0000000000000507

**Published:** 2020-08-18

**Authors:** Ivanna M. Pavisic, Jennifer M. Nicholas, Antoinette O'Connor, Helen Rice, Kirsty Lu, Nick C. Fox, Natalie S. Ryan

**Affiliations:** From the Department of Neurodegenerative Diseases (I.M.P., J.M.N., A.O., H.R., K.L., N.C.F., N.S.R.), Dementia Research Centre, UCL Queen Square Institute of Neurology, London; UK Dementia Research Institute at University College London (I.M.P., A.O., H.R., N.C.F., N.S.R.); and Department of Medial Statistics (J.M.N.), London School of Hygiene and Tropical Medicine, United Kingdom.

## Abstract

**Objective:**

To use survival modeling to estimate disease duration in autosomal dominant familial Alzheimer disease (ADAD) and ascertain whether factors influencing age at onset also affect survival.

**Methods:**

Symptomatic mutation carriers (201 presenilin 1 [*PSEN1*] and 55 amyloid precursor protein [*APP*]) from ADAD families referred to the Dementia Research Centre, between 1987 and 2019, were included. Survival was assessed with respect to age at onset, year of birth, *APOE* ε4 status, cognitive presentation, and sex using multilevel mixed-effects Weibull survival models. The contribution of mutation and family to variance in age at onset and duration was also assessed.

**Results:**

Estimated mean survival was 11.6 (10.4–12.9) years and was similar for *APP* and *PSEN1* mutations. Sixty-seven percent of the variance in age at onset was explained by mutation and 72% by mutation and family together. In contrast, only 6% of the variance in disease duration was explained by mutation specificity and 18% by family membership. Irrespective of gene, survival appeared longer for successive generations and in individuals with atypical presentations. Older age at onset was associated with longer duration within *PSEN1* and shorter duration within *APP* mutation carriers. No differences in survival time were found between sexes or between mutations located before or beyond codon 200 within *PSEN1*.

**Conclusions:**

Survival is influenced by mutation to a much lesser extent than age at onset. Survival time has increased over time and is longer in atypical presentations. These insights may inform the interpretation of disease-modifying therapy trials in ADAD.

There are currently no disease-modifying treatments for Alzheimer disease (AD). Although the search for such treatments continues, it is relevant to investigate variability in disease duration and to study factors influencing survival time.^1^ Autosomal dominant familial Alzheimer disease (ADAD) accounts for less than 1% of all AD cases.^2^ Pathogenic mutations in presenilin 1 (*PSEN1*),^3^ presenilin 2,^4^ or amyloid precursor protein (*APP*)^5^ are nearly 100% penetrant, and age at onset is correlated among family members.^6^ This offers a unique opportunity to study survival after symptom onset relatively precisely. Mean age at onset tends to be significantly later for *APP* than *PSEN1* mutations,^6^ and a previous study of our *PSEN1* cohort found that 72% of the variance in age at onset was explained by the specific mutation and 82% by mutation and family membership together.^7^ As in sporadic AD (particularly in young-onset AD),^8^ some patients present atypically with initial symptoms involving cognitive domains other than memory (e.g., language or behavior^7,9^), which tend to be more common in *PSEN1* compared withh *APP*, and particularly in *PSEN1* mutations beyond codon 200.^7^ Given that a considerable amount of the variability in age at onset can be explained by genetic factors,^6^ we undertook the current study to investigate the hypothesis that genetic differences also affect disease duration in ADAD.

Previous studies have often estimated disease length, including only patients who have died, by subtracting an individual's age at onset from their age at death. This leads to an intrinsic bias against longer disease durations as individuals who are affected, but have not yet died, cannot be included.^10^ There have only been a few comprehensive studies of age at onset and disease course in ADAD, including a meta-analysis,^6,11,12^ but none of these used survival models to account for censoring in date of death in those who did not die during the follow-up period. Investigations into generational effects on survival time in ADAD are also lacking, and survival modeling may offer a useful approach to evaluate these issues.

The current study addresses differences in survival time between *APP* and *PSEN1* mutations, *APOE* ε4 carriers and ε4 noncarriers, sexes, cognitive presentations, and *PSEN1* mutation position in relation to codon 200. We also evaluate the extent to which disease duration varies by mutation and family within each gene and the influence of age at onset and year of birth on disease duration. We report these differences in detail while accounting for censoring.

## Methods

### Study design and participants

We conducted a retrospective cohort study of families with histories suggestive of ADAD, which were referred to the Dementia Research Centre at University College London's Institute of Neurology (London, United Kingdom) from clinical and research centers across the United Kingdom and Ireland between July 1, 1987, and September 2, 2019. We used clinical and genetic data from these families (table 1). Inclusion criteria for the study were a family history suggestive of ADAD and known age at symptom onset. Exclusion criteria were a neurodegenerative condition other than ADAD, unknown age at symptom onset, unknown year of birth, and no information on last year of contact with the center.

**Table 1:**
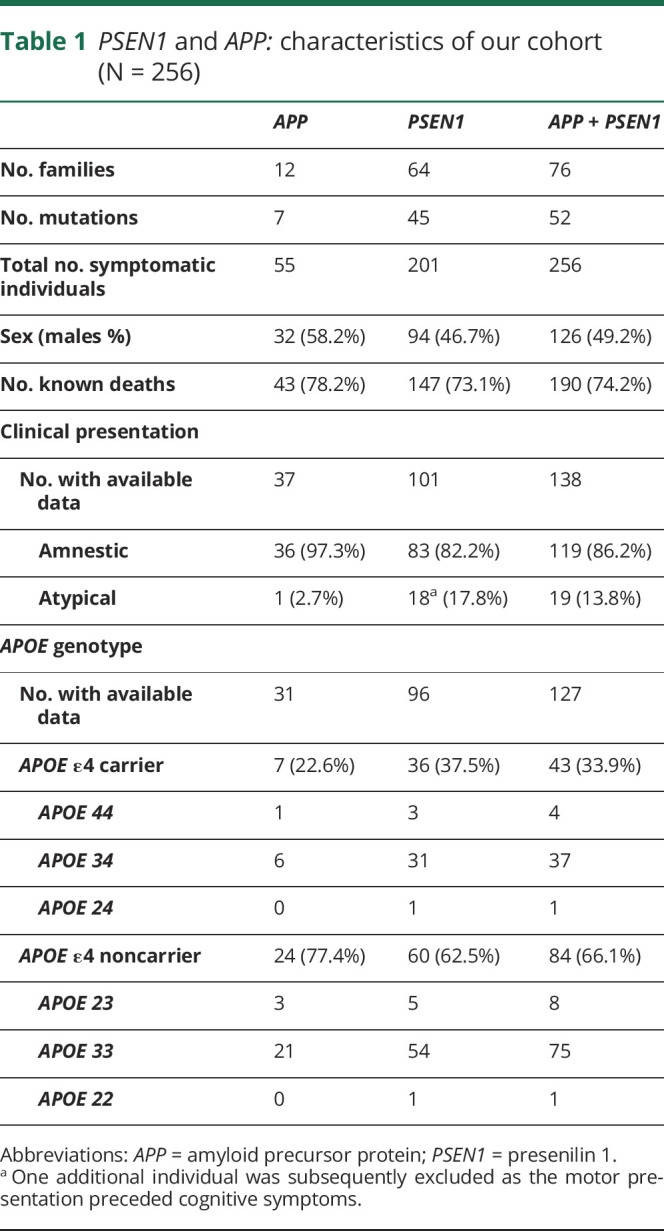
*PSEN1* and *APP:* characteristics of our cohort (N = 256)

### Procedures

Contemporaneous records were evaluated to determine age at onset, defined as the age at which progressive symptoms of cognitive, behavioral, or motor changes were first noticed by someone who knew the patient well, and the nature of the initial symptoms. The cognitive presentation was classified as either amnestic, for those with initial memory symptoms, or atypical, for those with nonamnestic initial symptoms such as behavioral change or symptoms of language or executive dysfunction or dyscalculia. One individual was excluded from the cognitive presentation analysis as they had a motor presentation that preceded cognitive symptoms. Age at death was ascertained from examination of medical records, postmortem reports, and interviews with living relatives and was collected up to September 2019. Disease duration was calculated by subtracting the age at death from the age at onset where this was available (N = 197), and where this was not available, the disease duration at censoring was calculated from the age of the individual at their last assessment (N = 71). One participant with 2 *PSEN1* substitutions (p.Thr291Ala and p.Ala343Thr) was excluded from the exon analysis because it was unclear whether pathogenicity was due to one or both of these amino acid substitutions.^7^ Twelve additional individuals were excluded from all analysis: 5 due to uncertainty in year of last contact (information necessary for censoring) and 7 due to unknown year of birth (variable considered as a covariate in all models) (figure e-1, supplementary materials, links.lww.com/NXG/A312). The intron 4 mutation was classified as involving exon 4 because it is located just outside this exon.

Mutation analysis was performed as described previously^7,13^ using Sanger sequencing. *APOE* ε4 status was determined by the Medical Research Council (MRC) Prion Unit (London, United Kingdom) using minor groove binding probe genotyping assays (TaqMan, Applied Biosystems). As described in our previous work, individuals with novel variants in *PSEN1* or *APP* were assessed for the presence of additional mutations in other dementia-related genes using the MRC Dementia Gene Panel.^7,14^ All novel sequence variants were absent from the Genome Aggregation Database (gnomad.broadinstitute.org/).

In total, 256 individuals were included in the analyses (201 with *PSEN1* and 55 with *APP* mutations) (table-e1, supplementary materials for specific mutations, links.lww.com/NXG/A312).

### Statistical analysis

We investigated differences in disease duration between *APP* and *PSEN1* genes, *APOE* ε4 carriers and ε4 noncarriers within each genetic group, cognitive presentation, sex, exon number, and position in relation to codon 200 (*PSEN1* only) using the Kaplan-Meier survival estimate for descriptive statistics and Weibull multilevel parametric survival analysis (using an accelerated failure time model) to compare the survival function of different groups of patients and test the specific hypothesis. Following the second-order relationship between disease duration and age at onset in Ryman et al.^6^ meta-analysis, we predefined that we would investigate a quadratic term for age at onset. We tested the interaction with gene. Sex, year of birth (range: 1879–1983), and gene were included as fixed effects and family (as a proxy to mutation) as random effects in all survival models. The intraclass correlation coefficient (ICC) was used to quantify the proportion of variance in disease duration explained by mutation and family (supplementary materials, links.lww.com/NXG/A312).

Linear mixed-effects models with random effects for mutation and family and fixed effects for sex, year of birth, and gene were used to compare differences in age at onset between genes and cognitive presentations within *PSEN1* mutations.

We used *p* < 0.05 as our measure of statistical significance and Stata v14 (StataCorp 2015) or later for all analyses. Bonferroni correction for multiple comparisons was applied for comparison of disease duration between exons.

### Standard protocol approvals, registrations, and patient consents

The study was approved by The National Hospital for Neurology and Neurosurgery and Institute of Neurology Joint Research Ethics Committee (subsequently, National Research Ethics Service Committee, London Queen Square, Research Ethics Committee ref 11/LO/0753). Written informed consent was obtained from all participants or from their consultee if cognitive impairment prohibited written informed consent.

### Data availability

Anonymized data will be shared by request from any qualified investigator.

## Results

Age at symptom onset was available for all 256 individuals included (201 with *PSEN1* and 55 with *APP* mutations). Age at death was available for 190 of those individuals (77.0% of the data set: 147 *PSEN1* and 43 *APP* mutations) (table 1).

### Disease duration and age at onset: *APP* vs *PSEN1*

**Figure 1:**
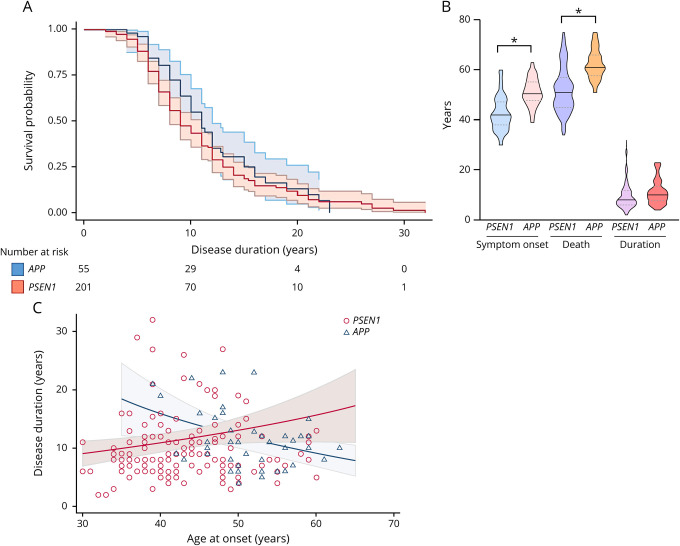
Symptom onset, age at death, disease duration, and survival probability by gene (A) Unadjusted Kaplan-Meier survival plots show the estimated survival probability by disease duration for *PSEN1 vs APP*. The blue line references *APP* and the red line *PSEN1*. Ninety-five percent CIs and number of individuals still alive per disease duration length, by 10 years, by 20 years, and by 30 years, are also shown. (B) Violin plots show the distribution of age at symptom onset, at death, and disease duration for *PSEN1 vs APP*. Data are median (line) with median interquartile range (upper and lower dotted lines). Age at onset: 42 (38–48) years vs 50 (48–55) years; age at death: 52 (46–58) years vs 61 (58–66) years; and disease duration: 8 (6–12) years vs 10 (8–13) years. “*” indicates significant difference between groups. (C) Scatter plot shows the association between age at symptom onset and age at death in *PSEN1 vs APP*. The solid line represents the line of best fit from the survival model, adjusted for sex, year of birth, and clustered by family membership for each gene. The shaded area represents 95% CIs. Markers show the unadjusted raw data: hollow blue triangles represent individuals with *APP* mutations and hollow red circle markers individuals with *PSEN1* mutations. *APP* = amyloid precursor protein; CI = confidence interval; *PSEN1* = presenilin 1.

Considering only individuals with known age at death (which does not take into account censoring) (N = 190), the mean disease duration was 10.4 years (SD 5.3), range: 2–32 years. Survival analysis (N = 256) revealed a 75% probability of surviving at least 7 years, 50% of surviving at least 10 years, 25% of surviving at least 14 years, and an estimated mean duration of 11.6 (10.4–12.9) years. Estimated survival time was similar between *APP* and *PSEN1* mutation carriers (table 2, figure 1).

**Table 2:**
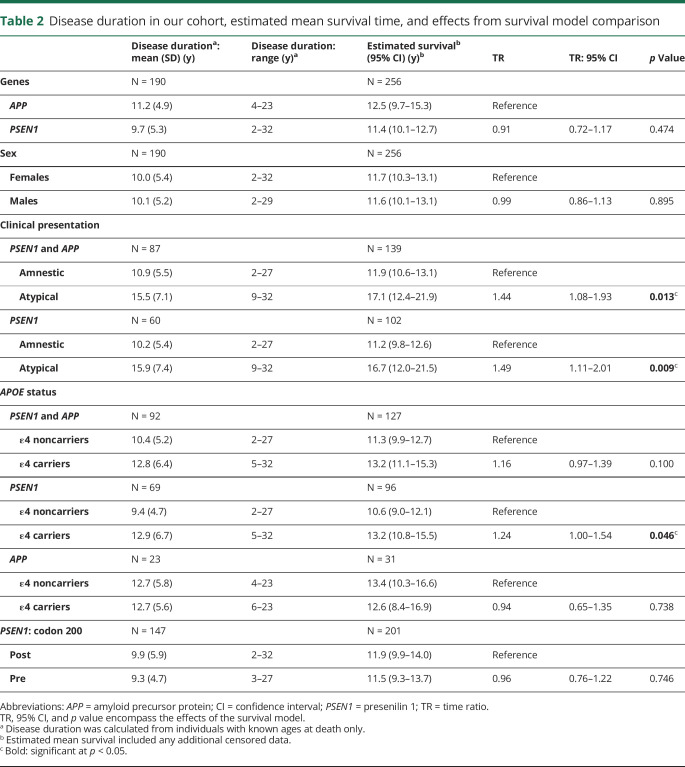
Disease duration in our cohort, estimated mean survival time, and effects from survival model comparison

Considering the cohort as a whole, family membership explained 18% (ICC 0.18; *p* < 0.001) of the variability in disease duration, and mutation specificity explained 6% (ICC 0.06; *p* = 0.188). In patients with a *PSEN1* mutation, 25% of the variance in disease duration was explained by family membership (ICC 0.25, *p* < 0.001) and 10% by a specific mutation (ICC 0.10, *p* = 0.129). Data were not analyzed separately for *APP* mutations due to small numbers.

In accordance with our previous work,^7^ age at onset was on average 7.1 years later for individuals with *APP* mutations (mean age 50.6 years [SD 5.6], range 38–63 years) than those with *PSEN1* mutations (43.5 years [7.2], range: 30–62 years; *p* < 0.001) (figure 1) (figure e-2, links.lww.com/NXG/A312, for age at onset distribution of sample). In patients with *PSEN1* mutations, 72% of the variance in age at onset was explained by mutation (ICC 0.72, *p* < 0.001). Mutation and family membership together explained 80% of the variance in age at symptom onset (ICC 0.80, *p* < 0.001). Considering both genes together, 67% of the variance was explained by mutation and 72% by mutation and family together.

Although no linear relationship between an individual's age at onset and the estimated length of disease course was observed {time ratio [TR] = 1.00 (95% confidence interval [CI] 0.99–1.01), *p* = 0.286}, there was an interaction between age at onset and gene in relation to estimated survival (TR = 1.05 [1.02–1.08], *p* = 0.001). In *PSEN1* mutations, later ages at onset were associated with longer disease durations (disease duration increased by 1.8% for every 1-year increase in age at onset [95% CI 0.3–3.4]). Whereas in *APP*, later ages at onset were associated with shorter disease durations (disease duration decreased by 3.0% for every 1-year increase in age at onset [95% CI 0.9–4.7]) (figure 1). Like Ryman and colleagues,^6^ we detected an “inverted-U” shape relationship between age at onset and age at death such that patients with early (younger than 40 years) or late (older than 50 years) onset each had shorter disease duration than patients with onset in midlife (40–50 years) irrespective of the gene (χ^2^ = 6.12, *p* = 0.047; considering age at onset as a quadratic term). However, including the gene interaction abolished this quadratic association (χ^2^ = 1.33, *p* = 0.515), indicating that gene membership may have driven the “inverted-U” shape effect.

### Sex and year of birth

Sex did not appear to affect disease duration, either for the cohort as a whole (table 2) or for genes separately (data not shown).

**Figure 2:**
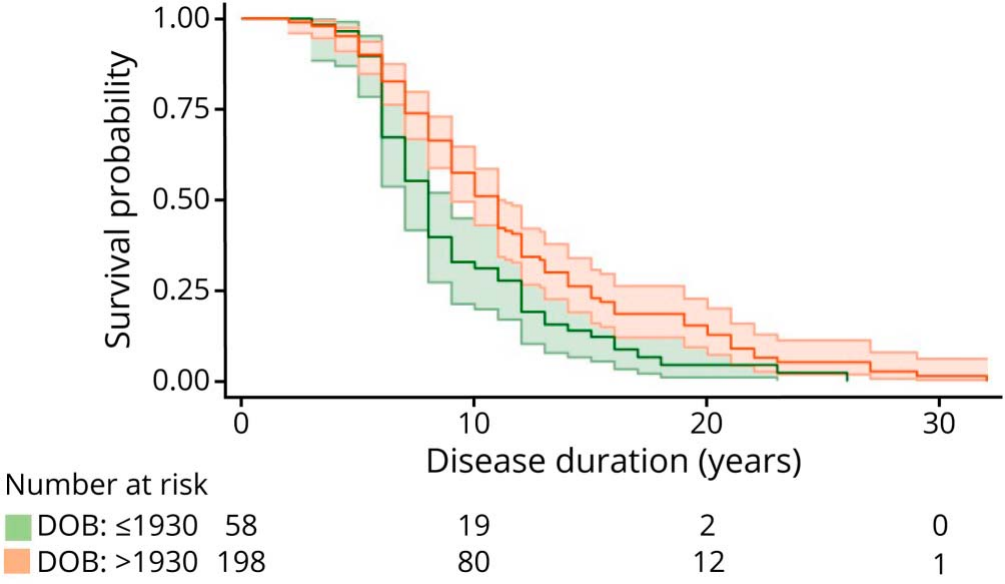
Survival probability pre- and post-births in the 1930s Unadjusted Kaplan-Meier survival plot showing survival by disease duration for individuals born before and after the 1930s. Green references individuals born by 1930 and orange after 1930. Ninety-five percent CIs and number of individuals still alive per disease duration length, by 10 years, by 20 years, and by 30 years, are also shown. CI = confidence interval; DOB = date of birth.

Irrespective of the gene, an individuals' year of birth appeared to influence survival and age at onset, with age at onset being earlier and duration longer in more recent times. Disease duration increased by 0.6 (95% CI 0.2–1.0) % for every increase in 1 year of birth (*p* = 0.003). This was also the case when considering genes separately. Further analysis revealed that the greatest difference in survival time was between individuals born before and after 1931: estimated survival: 9.1 (7.7–10.4) years vs 12.2 (10.8–13.5) years (figure 2). Age at onset decreased by 0.04 (0.01–0.07) years for every increase in 1 year of birth (*p* = 0.004). Comparing individuals born before or after the 1930s, we found that the average age at onset was 45.5 (43.3–47.7) years vs 44.6 (42.7–46.5) years, respectively (estimated difference = 0.9 [−2.1 to 0.4] years, *p* = 0.181). The significantly longer survival time for later generations remained after adjusting for age at onset (0.6 [0.2–1.0] % increase in survival for every increase in 1 year of birth; survival estimates pre vs post births in the 1930s: 8.9 [7.6–10.3] years vs 12.2 [10.9–13.5] years).

### Clinical presentation

**Figure 3:**
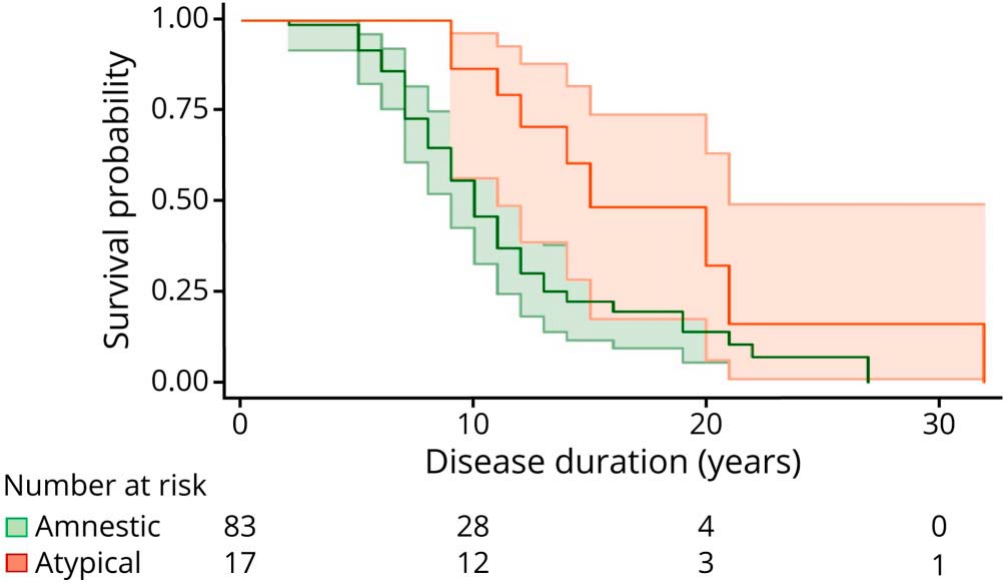
*PSEN1*: survival probability by clinical presentation Unadjusted Kaplan-Meier survival plot shows the estimated survival probability by disease duration for clinical presentations. Ninety-fivepercent CIs and number of individuals still alive per disease duration length, by 10 years, by 20 years, and by 30 years, are also shown. Green references individuals with amnestic presentations and orange individuals with atypical presentations. CI = confidence interval; *PSEN1* = presenilin 1.

The *PSEN1* subgroup with atypical cognitive presentations had, on average, a somewhat older age at onset than those with *PSEN1* carriers with amnestic presentations (amnestic: 42.4 years [SD 7.3)] range: 30–62 years vs atypical: 45.4 years [5.7], range: 38–58 years), but there was little evidence to support this difference, *p* = 0.592) (figure e-3, supplementary materials, links.lww.com/NXG/A312). Within the *PSEN1* group, individuals with atypical presentations had a 49.2% longer survival time compared with those with amnestic presentations (table 2 and figure 3). Only 8% of the variance in survival time between individuals with the same cognitive presentations in *PSEN1* mutations was explained by family membership (ICC 0.08, *p* = 0.157). The difference in estimated survival time between cognitive presentations was replicated combining *APP* and *PSEN1* groups together (table 2). We did not find any interactions between the cognitive presentation and codon 200 position, *APOE* ε4 status, or age at onset (data not shown). Similar results emerged when we included age at onset as a covariate in the model.

### *APOE* ε4 status

*APOE* ε4 status did not have an effect on age at onset in our cohort (mean [95% CI]: ε4 carriers = 44.0 [41.8–46.1] years vs ε4 noncarriers = 44.5 [42.5–46.5], *p* = 0.495). Considering *PSEN1* and *APP* mutation carriers together, survival analysis (N = 127) revealed similar estimates between ε4 carriers and ε4 noncarriers (table 2).

There was no interaction between *APOE* ε4 status and gene (TR = 1.35 [0.87–2.08], *p* = 0.180). Nonetheless, further analysis revealed an effect seemingly restricted to *PSEN1* mutations (N = 96): there was some evidence that ε4 carriers had longer survival time compared with ε4 noncarriers (table 2). *APOE* ε4 status did not have an effect on disease duration in the small group of individuals with *APP* mutations (N = 31) (table 2) (figure e-5, supplementary materials, links.lww.com/NXG/A312). We then examined *APOE* ε4 heterozygous and homozygous groups separately. Because of statistical power limitations for homozygous carriers (N = 4), we report heterozygous ε4 carrier results only (N = 38). In the whole cohort, there was a trend toward carriers of 1 ε4 allele having a 20% longer survival time compared with ε4 noncarriers (13.7 [11.3–16.0] years vs 11.4 [10.0–12.9] years, *p* = 0.056). Within the *PSEN1* cohort, the possession of 1 ε4 allele was associated with a 30% longer survival time (13.7 [11.1–16.3] years vs 10.6 [9.0–12.1] years, *p* = 0.023). Comparing carriers of 1 ε4 allele with noncarriers in the *APP* cohort did not reveal any differences (12.3 [7.4–17.3] years vs 13.5 [10.1–17.0] years, *p* = 0.677) (figure e-6 and table e-2, supplementary materials, links.lww.com/NXG/A312). Considering age at onset in these models did not change results (data not shown).

### *PSEN1* mutation location

Survival time did not differ between individuals with *PSEN1* mutations located pre- or post-codon 200 (table 2). Considering age at onset in these models did not change results (data not shown). Some individuals with *PSEN1* mutations in exon 8 (N = 40) appeared to reach particularly long disease durations (mean exon 8 duration—in those with known age at death: 11.3 years [SD 5.9], range: 5–32 years; figure e-4, supplementary materials, links.lww.com/NXG/A312). After adjusting for multiple comparisons (28 comparisons: Bonferroni correction), mutations located in exon 8 (N = 58) had longer survival estimates than in those in exon 11 (N = 6) (14.0 [10.8–17.2] years vs 6.2 [3.4–9.0] years, *p* = 0.034). Fifteen percent of the variability in survival time among those with a mutation on the same exon was explained by family membership (ICC 0.15, *p* = 0.004).

## Discussion

In this study, individuals with *APP* mutations had, on average, similar estimated survival time to individuals with *PSEN1* mutations—despite the *APP* group having an age at onset that was, on average, 7 years later than the average age at onset in the *PSEN1* group. Estimated mean survival for our cohort was just over a decade. There was, however, great variability in disease duration for both the *PSEN1* (2–32 years) and *APP* (4–23 years) groups and unlike age at onset, mutation type, and family membership explained relatively little of this variance. In this respect, it may be relevant that we found family membership to account for a slightly larger proportion of variance in disease duration than mutation type, although shared environmental factors could also contribute to this finding. Although longer survival for females compared with males has been reported in sporadic AD,^15^ our results do not provide evidence for sex differences in survival in ADAD, which affects individuals at a much younger age.

In accordance with Ryman et al.^6^ meta-analysis, there was a trend for longer disease duration in individuals with an age at onset of 40–50 years (compared with <40 years or >50 years). Looking at *PSEN1* and *APP* mutation carriers separately suggested that while in *PSEN1* mutations, later ages at onset were associated with longer disease durations, in *APP* later ages at onset were associated with shorter disease durations. Although it is unclear why these differences between *APP* and *PSEN1* exist, different paths of disease course between genes may underly this “inverted-U” shape relationship observed also in other studies.^6^

Our results indicate that individuals born after 1930 had longer survival time compared with those born in previous generations and that age at onset was earlier with more recent years of birth. These suggest that gradually (with no step change), onset or recognition of onset has come earlier. This may likely be due to greater awareness within families, with onset coming about 2 years earlier over the course of 2 generations (∼50 years). Nonetheless, survival has increased over and above this. As the difference in age at onset between births before and after 1931 was smaller than the difference in survival time (0.9 years vs 3.1 years), this increase in survival time could not solely be explained by earlier awareness of symptoms. The increase in survival observed over the study period accords with the fact that care, as well as life expectancy, has improved. Notably, antibiotics would have become widely available by the time individuals born after the 1930s were clinically affected.^16^

Despite phenotypic and pathologic differences reported between *PSEN1* mutations located before and beyond codon 200^7,17,18^ we,^19^ like others,^17^ found that disease duration did not significantly differ between these mutation groups. Atypical presentations have been reported to be more common with *PSEN1* mutations beyond codon 200 in our cohort, and the prevalence of atypical symptoms also differs markedly between exons, with nonamnestic cognitive presentations and pyramidal signs particularly common with mutations located in exon 8.^7^ Findings from the current study suggest that individuals with exon 8 mutations may also have particularly long disease durations. An intronic polymorphism in PSEN1 between exon 8 and exon 9 has been reported to show a significant association with late-onset disease.^20,21^ There may be differences in the disease process induced by variants located in this region of *PSEN1*, which drive later ages at symptom onset, longer disease durations, and atypical presentations.

Our findings suggest that carrying *APOE* ε4 may be associated with increased survival time in individuals with *PSEN1* mutations, but not in *APP* mutation carriers. However, this would need confirmation as we were not able to demonstrate a significant difference between the 2 genetic groups in the effect of *APOE* ε4. Of interest, the rare *APOE* ε3 Christchurch p. Arg136Ser mutation has recently been reported to delay onset of cognitive symptoms by 3 decades in a carrier of the Colombian *PSEN1* p.Glu280Ala mutation.^22,23^ These findings could have implications for the role of *APOE* in the pathogenesis, treatment, and prevention of AD and highlight how much remains unknown about the complexities of interactions between different genetic risk factors and their influence on disease onset and survival. Larger ADAD studies that consider the full range of *APOE* genotypes and follow individuals over time are needed to untangle the multifaceted effects of the *APOE* genotype.

Our study has a number of limitations. First, we included individuals born over a range of 100 years. Although this brought the strength of allowing us to study generational effects, it may somewhat limit how much our findings on average disease durations may be generalized to newly diagnosed patients. Although our analysis was adjusted for year of birth, replication in larger cohorts of more recently diagnosed individuals is needed. Second, we were not able to consider the effects of lifestyle (e.g., exercise) or life course (e.g., socioeconomic position) factors on survival. Future investigations should study the potential influence of lifestyle elements on survival rates, particularly in light of our finding that genetic factors contribute relatively little to the variance in disease duration we observed in our cohort. This is especially important to better understand the generalizability of our findings to individuals in other ADAD and sporadic AD cohorts. Third, cognitive presentations were classified as atypical on the basis that the initial symptoms did not involve memory but instead comprised behavioral change, language impairment, dyscalculia, or executive impairment. Atypical symptoms are often more difficult to recognize as signs of AD, leading to a possible underrepresentation of this group. Nonetheless, it is perhaps noteworthy that the atypical group had a longer disease duration, despite the possibility that symptoms may be noticed later, supporting the notion that there may be biological differences in those with atypical presentations, which underpin both the atypical presentation and the longer disease durations. Last, although we have included a relatively large number of cases, considering the rarity of ADAD, the sample size could be considered a limitation and further investigation of survival in larger ADAD cohorts will be an important direction for future research.

Multiple factors may contribute to phenotypic variability in ADAD. Characterizing and investigating variability in disease duration is the first step toward allowing patients and their families to plan for the future. A deeper understanding of variability in disease duration and the factors that may influence survival may also inform the interpretation of disease-modifying trials and potentially even highlight new avenues for targeting the disease.

## Glossary

AD: Alzheimer disease
ADAD: autosomal dominant familial Alzheimer disease
*APP*: amyloid precursor protein
CI: confidence interval
ICC: intraclass correlation coefficient
MRC: Medical Research Council
*PSEN1*: presenilin 1
TR: time ratio
